# COVID-19 Self-Reported Symptom Tracking Programs in the United States: Framework Synthesis

**DOI:** 10.2196/23297

**Published:** 2020-10-22

**Authors:** Tracey Pérez Koehlmoos, Miranda Lynn Janvrin, Jessica Korona-Bailey, Cathaleen Madsen, Rodney Sturdivant

**Affiliations:** 1 Uniformed Services University Bethesda, MD United States; 2 Health Services Research Program Henry M Jackson Foundation for the Advancement of Military Medicine Bethesda, MD United States

**Keywords:** COVID-19, coronavirus, framework analysis, information resources, patient-reported outcome measures, self-reported, surveillance, monitoring, symptom tracking, synthesis

## Abstract

**Background:**

With the continued spread of COVID-19 in the United States, identifying potential outbreaks before infected individuals cross the clinical threshold is key to allowing public health officials time to ensure local health care institutions are adequately prepared. In response to this need, researchers have developed participatory surveillance technologies that allow individuals to report emerging symptoms daily so that their data can be extrapolated and disseminated to local health care authorities.

**Objective:**

This study uses a framework synthesis to evaluate existing self-reported symptom tracking programs in the United States for COVID-19 as an early-warning tool for probable clusters of infection. This in turn will inform decision makers and health care planners about these technologies and the usefulness of their information to aid in federal, state, and local efforts to mobilize effective current and future pandemic responses.

**Methods:**

Programs were identified through keyword searches and snowball sampling, then screened for inclusion. A best fit framework was constructed for all programs that met the inclusion criteria by collating information collected from each into a table for easy comparison.

**Results:**

We screened 8 programs; 6 were included in our final framework synthesis. We identified multiple common data elements, including demographic information like race, age, gender, and affiliation (all were associated with universities, medical schools, or schools of public health). Dissimilarities included collection of data regarding smoking status, mental well-being, and suspected exposure to COVID-19.

**Conclusions:**

Several programs currently exist that track COVID-19 symptoms from participants on a semiregular basis. Coordination between symptom tracking program research teams and local and state authorities is currently lacking, presenting an opportunity for collaboration to avoid duplication of efforts and more comprehensive knowledge dissemination.

## Introduction

### Background

A 2019 outbreak of febrile respiratory illness in Wuhan, China, quickly evolved into the COVID-19 pandemic [1]. The disease has affected over 200 countries and territories worldwide. Globally, there are more than 18 million confirmed cases and over 700,000 deaths attributed to this flu-like illness, as of August 6, 2020 [2]. In the United States alone, there are more than 4.5 million confirmed cases and over 150,000 deaths [3]. The true number of those affected may be much higher due to the slow rollout and lack of availability of testing in the United States compared to other countries [4].

The United States, as well as other countries, has combatted this pandemic and sought to flatten the curve via social distancing, testing, isolation, and contact tracing [5]. Despite best efforts, the virus spread quickly with serious implications. In the first month of testing, the hospitalization rate was 4.6 per 100,000 people in the United States. Hospitalization rates were highest among adults over 65 years as well as those with underlying conditions [6]. At the present time, there is no specific antiviral treatment for COVID-19. Management of symptoms focuses on supportive care and oxygen therapy, both of which involve a plethora of hospital resources [5]. Modeling of COVID-19 shows that the pandemic has the potential to cause regional shortages of hospital beds, intensive care unit (ICU) beds, ventilators, and medical staff, which could lead to difficult ethical decisions [7]. A recent study suggests that COVID-19 will likely become endemic like cold and flu viruses [8]. There is a need to predict where resources should be distributed before potential patients with COVID-19 enter the hospital setting to alleviate strain on medical staff and facilities.

Epidemiological surveillance is fundamental in coordinating both immediate and long-term strategies for the detection and prevention of infectious disease outbreaks [9,10]. However, since collecting and disseminating these data take several weeks, during highly transmissible outbreaks, it may not be entirely reflective of the current prevalence of the disease. As these data are used to inform health authorities and prompt a public response, the resulting time delay can lead to inappropriate or inadequate response to actual need. Additionally, the collected data may be incomplete or insufficient to discern regional demographics that may impact effective intervention and treatment [11,12].

To overcome the limitations of epidemiological surveillance, internet-based technologies have been developed to estimate and monitor real-time changes in population, soliciting participation from the public at large [12,13]. One approach that has been introduced is self-reported symptom tracking. Symptom tracking is a form of crowd-sourced participatory surveillance that solicits individuals to report their health status on a daily or weekly basis, often with emails or notifications to prompt timely response, allowing researchers to see potential changes in the population before seeing changes in clinical presentation at hospitals and medical centers. Symptom tracking is used primarily to track and forecast influenza activity throughout the country; however, researchers have been looking to apply this technology to other diseases, such as COVID-19 [12,13]. Participatory surveillance such as this may prove vital to complement epidemiological surveillance during highly transmissible epidemics, as it allows for the detection of outbreaks before they reach the clinical threshold, affording more time for logistical support and appropriate allocation of resources [11]. Research by Baltrusaitis et al [13] indicates that collected participatory surveillance of influenza later correlated with confirmed epidemiological surveillance data. With the current highly transmissible and deadly COVID-19 pandemic putting a strain on portions of the United States health care system, participatory surveillance is more important than ever to bolster local prevention efforts [14].

### Research Purpose

This study uses a framework synthesis to inform decision making about the utility of existing self-reported symptom tracking programs for COVID-19, with a focus on the US population, as an early-warning tool for probable clusters of infection. Due to the rapidly changing nature of both the pandemic and work in this area, this research will be updated at 6- and 12-month intervals.

### Objective

The purpose of this framework analysis is to assess the number and scope of self-reported symptom tracker programs focused on the United States and COVID-19. An innovative best fit framework analysis was chosen because of its strength, utility, and appropriateness in drawing conclusions for an evolving subject [15,16]. According to Booth and Carroll [17], the best fit framework approach is considered a highly structured and pragmatic methodology for research synthesis suited for qualitative research with specific questions, a limited time frame, and issues that have been previously identified; this served the purpose of our research well [18]. The outcomes of this synthesis and its updates should inform decision makers and health care planners about these technologies and the information they can ascertain from them in order to aid in federal, state, and local efforts to combat the pandemic both now and in the future.

## Methods

A framework analysis was conducted to assess symptom tracking programs. A best fit framework was constructed by collating information collected from each program into a table for easy comparison between programs.

### Target Population

This framework synthesis sought to identify programs that track COVID-19 symptoms in the US population for all ages, genders, and ethnicities. Inclusion and exclusion criteria are shown below:

- Inclusion criteria: programs were included if they aimed to capture and geographically collate self-reported potential symptoms of COVID-19 and if they were available for use in the United States. For our purpose, a symptom tracking tool is defined as a program that allows individuals to report symptoms of COVID-19 to identify geographic areas with emerging or changes in progression of disease.
- Exclusion criteria: programs were excluded if they did not track specific symptoms for COVID-19, were symptom checkers for individual use only, or were not targeting the US population.

### Program Identification

Programs were identified using Google search for keywords (“symptom trackers covid,” “symptom trackers coronavirus,” “symptom tracking covid,” “symptom tracking coronavirus,” “daily symptom tracking covid,” “daily symptom tracking coronavirus,” “self-reporting covid,” “self-reporting coronavirus”). The time frame for the search for programs ranges from April 7, 2020, to May 9, 2020. Further, we used snowball sampling to identify other symptom tracker programs for COVID-19.

### Screening Method

Reviewers (JK, MJ, TK) screened programs to determine if inclusion criteria were met. Reviewers (MJ, JK, TK) then extracted data from program websites using a standardized form. To complete the collection of information not available via the program webpages, we contacted the managers of the programs via email.

### Synthesis Method

Data relating to program characteristics were extracted from all included programs and organized into a table format, which was used to guide data collection and build the framework for analysis. Data were then synthesized in order to form meaningful statements about the programs.

## Results

We identified 6 programs that met the inclusion criteria. Information was gathered from the public webpages of all eligible symptom trackers (BeatCOVID19Now, COVIDcast, COVIDNearYou, COVID Symptom Tracker, HelpBeatCOVID19, and HowWeFeel) (Table 1). Two programs, C19Check and the Department of Defense’s MySymptoms.mil, were excluded from our synthesis since they are symptom checkers that do not identify probable clusters of emerging infection.

All of the included programs were affiliated with a university, school of medicine, or school of public health. Half of the programs (n=3) included were based in Boston, Massachusetts, and affiliated with Harvard University (COVIDNearYou, COVID Symptom Tracker, and HowWeFeel), with COVIDNearYou also collecting data from participants in Canada and Mexico. Two other programs are based elsewhere within the United States (COVIDcast and HelpBeatCOVID19), and one is based in Australia, designed for international use (BeatCOVID19Now).

The number of responses, defined as unique symptom entries by an individual, to each program varied widely, with the lowest being ~27,000 (BeatCOVID19Now) and the highest being 2,573,240 (COVIDcast). COVID Symptom Tracker collected data from patients currently enrolled in large cohort studies and clinical trials not related to COVID-19 and had obtained much of their initial influx of responses through that mechanism. Two-thirds of the programs had fewer than 100,000 responses. Three programs utilized a website to collect data, while two exclusively used an app available for both Apple and Android devices (COVID Symptom Tracker and HowWeFeel), and only one utilized a survey on a social media platform (Facebook). While most of the programs had no form of follow-up with participants, COVIDNearYou and HelpBeatCOVID19 sent text message reminders, and COVID Symptom Tracker sent phone notifications every third day.

**Table 1:** Overview of self-reported symptom tracker programs.

Characteristic	BeatCOVID19Now	COVIDcast	COVIDNearYou	COVID Symptom Tracker	HelpBeatCOVID19	HowWeFeel
Host institution and partners	Swinburne University of Technology	Carnegie Mellon University Delphi Research Group; Facebook	Harvard Medical School; Boston Children’s Hospital; Ending Pandemics; Google; Centers for Disease Control and Prevention (CDC)	Harvard TH Chan School of Public Health; Massachusetts General Hospital; King's College London; Stanford University School of Medicine; Zoe Global Limited	University of Alabama; Alabama Department of Public Health	Harvard TH Chan School of Public Health; Massachusetts Institute of Technology; Institute for Quantitative Social Science; McGovern Institute; Howard Hughes Medical Institute; Weizmann Institute of Science; Pinterest; Feeding America; Alex’s Lemonade Stand; Chartio; Bill & Melinda Gates Foundation
Location	Melbourne, Australia	Pittsburgh, Pennsylvania, USA	Boston, Massachusetts, USA	Boston, Massachusetts, USA	Birmingham, Alabama, USA	Boston, Massachusetts, USA
Funding sources	Swinburne University of Technology	None	Ending Pandemics Crowdsourcing	Mass General Wellcome Trust (UK)	University of Alabama	Bill & Melinda Gates Foundation Crowdsourcing
Intended participants	Worldwide, 18+ years	United States residents, 18+ years	United States, Canada, and Mexico residents, 18+ years	United States residents, 18+ years; participants from other internal studies including RCTs	United States residents, 18+ years; particular focus on Alabama and neighboring states	United States residents, 18+ years
Date symptom tracker was initiated	March 26, 2020	April 6, 2020	March 22, 2020	April 4, 2020	Not available	April 3, 2020
Number of responses to date^a^	27,000+	2,573,240	54,000+	98,000+	57,000+	1,000,000+
Mechanism of recruiting participants/platform	Website; app in development^a^	Survey via Facebook	Website	Apple App Store, Google Play Store	Website	Apple App Store, Google Play Store
Follow-up	None	None	None	Daily phone notifications	Text messages every 3 days	None
Frequency of reporting	Daily	Daily	Weekly	Live data	Live data	Daily
Availability of summary tables for external synthesis/utilization	Yes	Yes	Yes	Yes	No	No^a ^
Intended audience for the product	Public at large; state and local public health officials; international health organizations	Public at large; state and local public health officials; US policymakers; health care providers; health care systems	Public at large; CDC and national public health organizations; state and local public health officials; researchers; health care providers; health care systems	Public at large; participants of internal studies	Public at large; neighboring states; state and local health officials; local policymakers	Public at large; state and local public health officials
Publicly available data privacy statement	Yes	Yes	Yes	Yes	Yes	Yes

^a^This data was collected at the time that the synthesis was performed and is subject to change.

The programs collected a variety of data elements, but several were common among them (Table 2). All of the symptom trackers collected demographic data on the participant’s age, gender, and zip code. They also all collected information on symptoms experienced by the participant, although the time frame considered varied from the present to 7 days prior. Additionally, every program asked if the participant had been tested for COVID-19 at the time of the survey. Five of the six trackers also asked for information on any chronic conditions that the participant is experiencing, and if they are or are not a smoker.

Some of the programs had special interest in certain topics that were not explored by others. Only four of the programs asked the participant if they had been exposed to anyone who had COVID-19, while two asked if the participant came into direct contact with the public. Two programs asked if participants had received an annual flu shot this past year. Two programs asked questions related to the impact of the pandemic on participant’s mental health. Two programs asked the participant to answer questions about others in their household in addition to themselves.

**Table 2:** Data elements across programs.

Data elements	BeatCOVID19Now	COVIDcast	COVIDNearYou	COVID Symptom Tracker	HelpBeatCOVID19	HowWeFeel
Is the survey being completed on behalf of another person?					✓	
Age	✓	✓	✓	✓	✓	✓
Gender/sex	✓	✓	✓	✓	✓	✓
Race/ethnicity	✓				✓	
Zip code	✓	✓	✓	✓	✓	✓
Number of people in the household	✓	✓			✓	
Employment status	✓					
Languages spoken in the household	✓					
Is the participant an essential worker?	✓					
Is the participant a health care worker?	✓			✓		
International travel within the past 2 months?	✓					
Has the participant traveled out of state within the past 5 days?		✓				
Travel within the past 2 weeks?			✓			
Does the participant come in direct contact with the public?				✓	✓	
How many people has the participant had direct contact with outside of their household?		✓				
Has the participant gone outside for work within the past 5 days?		✓				
What activities has the participant engaged in outside of their household?	✓					
Has the participant been in contact with health care professionals?			✓			
Has the participant visited a long-term care facility or nursing home within 5 days?		✓				
To what extent is the participant complying with social distancing guidelines?		✓				
How many days has the participant spent in quarantine or social isolation?			✓			
Has the participant been quarantined over the past 2 weeks?				✓		
Has the participant been quarantined over the past 24 hours?						✓
How is the participant feeling today? (good/not good)			✓	✓	✓	✓
Has the participant been exposed to anyone with COVID-19?		✓	✓	✓	✓	✓
Has the participant been tested for COVID-19?	✓	✓	✓	✓	✓	✓
Does the participant suspect they have COVID-19 despite not being tested?				✓		✓
Symptoms of the participant over the last 24 hours	✓					
Symptoms among the participant or household member(s) within 24 hours		✓				
Symptoms currently being experienced by the participant			✓	✓		✓
Symptoms over the last 7 days					✓	
How many days has the participant been experiencing symptoms?		✓				
What date did the participant begin experiencing symptoms?			✓			
Has the participant had difficulty completing normal activities over the past 24 hours?	✓					
Is anyone within the participant’s household experiencing symptoms?					✓	✓
Number of people in the household who are sick		✓				
Number of people the participant knows in the community who are sick		✓				
Has the participant been to the hospital within the past 24 hours?		✓				
Is the participant at home or hospitalized?				✓		
Is the participant able to move freely?				✓		
Does the participant require outside help on a regular basis?				✓		
If the participant needs help, can they get it from someone close to them?				✓		
Highest temperature		✓				
Does the participant have a non–COVID-19 respiratory illness?	✓					
Impact on immediate mental health or changes in mood or behavior	✓	✓				
Is the participant worried about their ability to engage in daily activities or about the security of their future?	✓	✓				
Does the participant have health problems that require staying indoors regularly?				✓		
Chronic conditions	✓	✓		✓	✓	✓
Smoking status				✓	✓	✓
Height				✓		
Weight				✓	✓	
Pregnancy status					✓	✓
Has the participant had the flu vaccination?	✓	✓	✓			
Is the participant currently taking aspirin?				✓		
Is the participant currently taking nonsteroidal anti-inflammatory drugs (NSAIDs)?				✓		
Is the participant currently taking blood pressure medication?				✓		
Is the participant currently taking immunosuppressants?				✓		
Does the participant have access to transportation?					✓	
Does the participant have health insurance?					✓	
Type of domicile					✓	
Can the participant afford a medical copay if needed?					✓	
Has the participant completed the survey before?					✓	

## Discussion

### Principal Results

Self-reported symptom trackers have been shown to be beneficial in tracking and monitoring the spread and progression of influenza each year and may prove to be vital as the United States continues to loosen shelter-in-place guidelines across the country. Due to the nature of the rapidly changing pandemic, this resource will be updated at both 6- and 12-month intervals to better reflect the evolving pandemic response.

Two of the programs were created by groups who already have existing infrastructure for tracking influenza outbreaks each year, BeatCOVID19Now, which is a derivative of Flu-iiQ, and COVIDNearYou, the sister tracker to FluNearYou. Flu-iiQ, in particular, was developed to solicit patient-reported outcome measures during large-scale clinical trials to measure the presence or absence of disease within a small subset of a population, allowing for extremely sensitive measurements without requiring thousands of responses [19]. The flexibility of these programs to track symptoms associated with diverse flu-like illness is imperative in identifying outbreaks of disease both for the purposes of this current pandemic as well as future flu and other respiratory disease outbreaks [20].

The data elements collected varied between programs, but all asked for zip code data, which means that even groups that do not currently have their data geolocated on maps have the potential to do so in the future in order to make data accessible to state and local health officials. They also all collect data regarding testing status, which enables local, state, or national program managers or planners to see the impact of current testing expansion efforts. Almost all of the programs asked about race and/or ethnicity, which may highlight racial disparities in testing, symptoms, unemployment status, and other chronic health conditions. The similarities in the data elements being collected by the different programs indicates that collaboration to build a larger, single picture is a possibility; standardization could be beneficial to the programs and to the local leaders and planners, health care providers, and researchers who would receive the outputs. The differences in collected data highlight areas of focus between the programs that other programs may want to consider incorporating as well.

Notable differences between the programs include unique data elements as well as the manner of recruitment. Two of the programs, BeatCOVID19Now and COVIDcast, are collecting information related to the mental health impact of the pandemic. This topic is currently being discussed in the scientific community since individuals with current mental health conditions can be at higher risk for infections [21,22]. Additionally, mental health conditions can be made worse by the anxiety and fear brought on by the pandemic [21]. Individuals without existing mental health conditions may develop emotional responses to the pandemic similar to disaster scenarios, particularly those who are working in response to the pandemic or those who are more susceptible to infection. Quarantine in general can spur a number of emotional responses that can remain after stay-at-home orders are lifted [22]. These programs could help to track the effect of mental health during the COVID-19 pandemic and help to inform prevention efforts for future pandemics requiring social isolation and quarantine. Another key difference was the reach of each program. Programs that partnered with or heavily relied on social media platforms (COVIDcast and HowWeFeel) had significantly more responses than those that did not utilize social media, suggesting that social media is a powerful recruitment tool for these efforts, even more so now since people depend on these platforms to stay connected due to social distancing measures. Therefore, its use should be considered by other groups going forward.

One of the notable results of this synthesis is the demonstrated overlap or duplication of effort between the programs. Each program is competing for the same group of potential respondents, who are more than likely going to be completing only one group’s survey. Without ongoing coordination between groups, the beneficiaries of their work—the public, lawmakers, state and local health care officials, etc—will not obtain information reflective of the full potential of symptom tracking. Although many of the groups recognize this, active collaboration between the groups has been a difficult process, even among the groups located in the same city (eg, Boston, Massachusetts) and based in the same institution.

A key challenge facing these programs is a lack of recognition at the national level. Only one of the trackers, COVIDNearYou, had a partnership with the Centers for Disease Control and Prevention, an extension of their ongoing partnership for FluNearYou. Despite this long-term collaboration, there is no outward support from the agency urging people to engage with this new program. The lack of local, state, or national promotion or outward partnership further exacerbates the potential for gaps between programs. Additionally, there is the potential that endorsement by local authorities or agencies could increase the number of responses, reaching people who were previously unaware of these programs and influencing them to contribute their data, which would in turn would allow for more complete data. This has been found to be the case in the United Kingdom, where the National Health System has endorsed the sister application to COVID Symptom Tracker, based at King’s College in London. Because of this, at the time of interview, they had received ten times as many responses as their US counterparts [23].

### Limitations

Several limitations must be acknowledged for this study. First, our analysis was limited to English language programs, and therefore may have missed nuances of data collection which are more important to non-English speaking residents. Second, although the speed of framework analysis enables rapid evaluation of commonalities, it does not provide the in-depth rigor of a full systematic review. Third, our collected data did evaluate differences in the number of responses to each program but not analyze the effectiveness, market penetration, or user demographics of evaluated programs. Fourth, we recognize that program participation is limited to only those who have access to the internet or cellular phone service, creating an unintended disparity among respondents based on their access to and utilization of technology. Therefore, the underlying reasons for the difference in response rate remain beyond the scope of this study. Last, this synthesis does not provide critical appraisal of programs or evaluate programs for effectiveness.

### Conclusion

Self-reported symptom tracking programs offer potential benefits as states and counties continue to reopen after the large-scale stay-at-home orders. Frequently reported data with high participation in geographic areas would allow officials to better monitor potential emerging hotspots and institute public health policy and reallocate resources more quickly to combat the spread of disease. However, there are unique challenges to address with self-reported symptom tracking programs to ensure successful implementation. Recognition or endorsement at the national, state, or local levels; increased funding to expand social media advertisements and partnerships; and collaboration between existing programs to generate a more comprehensive data picture would be essential steps in bolstering the utility of symptom tracking programs to achieve optimal effectiveness. If these challenges are addressed and symptom tracking programs become more widely used, the reopening process could be safer in the short term with the potential to monitor communities more closely for long-term management of the COVID-19 pandemic or future outbreaks.

## Abbreviations

CDC: Centers for Disease Control and Prevention
ICU: intensive care unit
